# 

**Published:** 1897-08

**Authors:** 


### NOTICES.

### Southwestern Michigan and Northern Indiana Dental Society.

The Bi-State Dental Meeting of the Southwestern Michigan and Northern Indiana Dental Society will be held at Benton Harbor, Tuesday and Wednesday, September 14 and 15, 1897.

F. H. ESSIG, *Secretary*, Michigan.
F. WEMER, *Secretary*, Indiana.

### Northern Iowa Dental Society.

The Third Annual Meeting of the Northern Iowa Dental Society will be held at Mason City, Iowa, September 7, 8 and 9, 1897. Arrangements already made indicate an interesting and profitable meeting.

A cordial invitation is extended to all members of the profession to meet with us.

WM. H. STEELE, *Secretary*,
Forest City, Iowa.

